# The Value of Cataphoresis

**Published:** 1902-11

**Authors:** Louis Jack

**Affiliations:** Philadelphia


### International Dental Journal.

VOL. XXIII. NOVEMBER, 1902. No. 11.

### Original Communications.[1]

### THE VALUE OF CATAPHORESIS.[2]

BY LOUIS JACK, D.D.S., PHILADELPHIA.

WHEN this subject was proposed at a previous meeting of this Academy, it was for the purpose of having the subject discussed in order to show the class of cases where cataphoric action is not required for the relief of dentinal sensitivity, and to determine where it is demanded. A further motive held in view is to promote the wider use of this beneficent means of relieving exquisite pain.

Before opening the subject in its practical relations, a summary or historical statement concerning the electrical diffusion of medicaments may be of interest.

The first investigator in this direction was Dr. B. W. Richardson, of London, who published two articles on Voltaic Narcotism in 1859. His experiments were conducted with aconite in combination with chloroform. With the aid of this combination he performed a series of successful operations, among them the extraction of five teeth. Here he applied a fine wire anode placed around

[1] The editor and publishers are not responsible for the views of authors of papers published in this department, nor for any claim to novelty, or otherwise, that may be made by them. No papers will be received for this department that have appeared in any other journal published in the country.

[2] Read before the Academy of Stomatology, Philadelphia, March 25, 1902.

the teeth and wound with cotton soaked in the above mixture, complete anæsthesia taking place in five minutes. Richardson met with intense opposition on the part of his medical *confrères,* for various reasons. He subsequently abandoned further experimentation, the development of general anæsthesia probably removing the incentive for further pursuit.

The subject remained dormant for over twenty-five years, when it was revived by Wagner, in 1886. Reynolds commenced to use cocaine in this way in 1887. Peterson began a series of interesting and conclusive experiments in 1888. In its application to dentistry the first published paper to appear was by B. F. McGrath, in 1888. Westlake read a paper before the New York State Dental Society in 1892, he claiming successful applications.

In 1895 Dr. Gillette presented before the American Dental Association an article containing explicit directions with relation to satisfactory experiments and description of operations, which enlisted immediate and general attention to the subject. It must be held that by his efforts this method of treatment was placed on a reliable basis.

Subsequently, in various papers, Dr. Price has demonstrated the scientific elements of this interesting procedure.

My experiments commenced in the early part of 1896, in which I followed the methods as disclosed by Dr. Gillette, but with a different apparatus. From that time to the present it has been in my hands a most reliable and certain means of giving relief in acute dentinal sensitivity. During this period I have administered this means over two hundred and sixty times, mostly in acute conditions. The result has been satisfactory, which I largely attribute to the apparatus I have employed,—namely, the dry cell chloride of silver battery, with an ampèremeter to measure the current strength, and the Willms controller to regulate the pressure to the varying degree of electric irritation presented.

The controller I first had in use was of one hundred thousand ohms at its greatest resistance. This was found in many cases to be insufficient. On the substitution of one of nominally four hundred thousand ohms the management of cases became extremely satisfactory. This individual controller, on being tested by Mr. Schramm, of the University of Pennsylvania, showed, at 70° F., at the first contact point a resistance of five hundred and eighty-nine thousand ohms. This degree of resistance with fifteen volts pressure is not extreme, as in a couple of cases of persons very susceptible to electrical irritation slight shock was manifest at the first contact point.

The apparatus here shown has been in use since April of 1896 without replenishment of the batteries or any repairs.

The cases where there is no need for the use of cataphoresis, as I would define them, are those of subacute sensitivity, when carbolic acid in combination with oil of cloves or caustic potash, or desiccation by heated air, will give relief. There are, however, many instances when sensitivity is so extreme that none of these means furnishes more than slight benefit. These compose the class of cases where, to excavate and shape the cavities, zinc chloride or general anæsthesia would be required. Zinc chloride generally is painful, is only applicable to shallow cavities, and should not be applied to the front teeth under any circumstances. Anæsthesia by ether is troublesome and questionable, except the person is sufficiently intelligent and sensible; therefore, when the permanent treatment of caries is necessary there is in my judgment no choice in the class under consideration outside of cataphoresis.

There are few persons who can in extreme sensitivity of dentine permit the careful preparation of cavities even after the slight modification which may be effected by the escharotics or by desiccation. These do not act deeply enough to be sufficiently effective.

It should be borne in mind that as an accompaniment of extreme dentinal sensitivity there nearly always exists high nervous irritability, which interdicts the infliction of pain. Here the simple requirements of human sympathy necessitate the avoidance of the consequences of operating without profound local anæsthesia.

The condition of the tooth after complete anæsthesia by cocaine cataphoresis enables the formation of the cavity to proceed without hinderance, and, except inconvenience of the situation may occur, the patient offers no resistance to the pursuit of the requirements of the case in hand. Furthermore, the nervous system of the patient escapes the strain otherwise imparted.

The effect of subjecting some persons to acute dental pain occasions shock, not apparent at the moment, but afterwards becomes manifest by the depression which follows the suffering.

It must be considered that too large a proportion of operative procedures are inefficiently carried out because of the inability of the patient to endure proper extension of the cutting and the formation necessary to secure sufficient retention. In some connection with this consideration is the effect produced upon the nervous force of the operator who endeavors to carry out these requirements without using the most efficient means of subduing acute sensitivity.

The objection has frequently been made of the time required to secure anæsthesia in this manner. This loss is more apparent than real, as, in the instances where cataphoric action is required, there is a real gain of time secured by the completion of the cases; whereas, with inefficient obtundation temporary fillings have to be inserted, thus indefinitely deferring permanent treatment.

Fortunately for operator and patient, the proportion of cases of acute dentinal sensitivity is small in comparison with those causing slight and moderate pain, but in my view, and from the results following cataphoresis, I have no question concerning the benefit in every respect following its use.

For the reasons herein given I am disposed to urge upon my colleagues to consider the subject anew, that the results of this benign mode of treatment may be further extended.

NOTE.—Much valuable information on the general subject of cataphoresis will be found in the "International System of Electro-Therapeutics," chaps. i., xx., by Frederick Peterson. The papers by Dr. Price in the various dental journals within the past four years will repay careful study.

