# Correction

**Published:** 1903-04

**Authors:** 


### CORRECTION.

In the article on "The Value of Cataphoresis, published in the November number of this journal, on page 782, the name of Dr. D. F. McGraw was incorrectly written B. F. McGrath in giving him credit for early experiments in the cataphoric administration of cocaine.

